# Using Novel Technology to Determine Mobility Among Hospitalized Heart Failure Patients: A Pilot Study

**DOI:** 10.4021/cr244w

**Published:** 2013-03-08

**Authors:** Jill Howie-Esquivel, Evanthia Zaharias

**Affiliations:** a Department of Physiological Nursing, University of California, San Francisco, San Francisco, USA; b Department of Case Management, UCSF Medical Center, San Francisco, CA, USA

**Keywords:** Immobility response, Heart failure, Accelerometer

## Abstract

**Background:**

Patients with heart failure (HF) experience frequent rehospitalizations and poor functional capacity. Early hospital mobility may prevent functional decline, but mobility patterns among hospitalized HF patients are not yet known. Accelerometers may provide a method to monitor and measure patient mobility objectively. Therefore, the purpose of this study was to describe mobility and function using accelerometers among hospitalized HF patients.

**Methods:**

Wireless accelerometers were attached to the thigh and ankle of previously ambulatory hospitalized HF patients (n = 32) continuously for up to 5 days, beginning on the second day of hospitalization. The mean proportion of time spent lying, sitting, and standing or walking daily was measured. Ability to perform activities of daily living (ADLs) and physical function was measured using the Katz Index and Short Physical Performance Battery (SPPB).

**Results:**

Patients’ mean age was 58.2 ± 13.6 and 78% (n = 25) were male. Mean New York Heart Association Class upon enrollment and at the end of the study period was 2.9 ± 0.8 and 2.2 ± 0.8 respectively. A mean Katz Index of 5.6 ± 1.1 upon enrollment demonstrated minimal dependence on assistance for completion of ADLs (possible scores 0 - 6). However, mobility testing revealed low physical function, with mean SPPB scores of 6.4 ± 3.1 (possible scores 0 - 12). During hospitalization, 70% of the measured hospital stay (16.8 hours/day) was spent lying in bed. The average time spent standing or walking was 4.1%, or 59 minutes per day and the range was 0-10% (0 - 150 minutes).

**Conclusions:**

Immobility was pervasive as HF patients spent almost all of their time sitting or lying in bed despite their baseline ambulatory status and improved NYHA class.

## Introduction

Bed rest, or immobility, has numerous deleterious effects such as muscle atrophy, deconditioning, and functional decline. In addition, immobility has been associated with increases in inflammatory markers and insulin resistance [1-4]. Immobilization can have multiple direct and indirect effects on musculature, even in healthy populations. During World War II the benefits of early ambulation on hospitalized soldiers was newly appreciated; morale was improved and recovery was faster so that troops could return to the battlefield more quickly [5]. Today, researchers continue to study the problems associated with immobility with one study reporting that older hospitalized adults spent most of their time lying in bed, despite their ability to ambulate independently before hospitalization [1].

The effects of immobility on patients hospitalized with heart failure (HF), a population with the highest hospital readmission rate in the US, are not known [6]. Underlying skeletal muscle atrophy coupled with diminished cardiac function accompany the worsening symptoms of dyspnea and fatigue when patients experience exacerbations of HF. Patients who experience volume overload while hospitalized for HF usually require aggressive intravenous diuretic therapies and are tethered to cardiac monitors, oxygen outlets, urine collection bags, and poles holding intravenous infusions. Signs and symptoms of peripheral edema and fatigue provide further challenges to ambulation.

The use of novel technologies, such as accelerometers, to monitor physical activity in older adults have gained widespread traction in research, but have not been used commonly in studies of hospitalized patients [7]. Direct observation and interviews or surveys have been used by researchers as the primary method to document patient activity in the hospital [7-9]. These subjective and resource-intensive methods are less than ideal when compared to the objective and accurate measurements that are associated with accelerometers [10]. Technology-based monitoring techniques to record physical activity and mobility include the use of pedometers and other sensors [11-13]. The monitoring systems are appealing in that they provide objective measurements non-invasively. However, the information provided, such as the number of steps walked, may not supply information on patterns or intensity of activity, or be accurate in frail or less active adults [13]. Accelerometers have the capacity to provide information about mobility that is very detailed, such as movement within a 3-dimensional axis [10]. With accelerometers, actual body position (lying, sitting and standing) can be continuously identified, stored and retrieved over the course of an entire hospitalization episode [10].

Hospital stays in older patients have been associated with loss of independence and physical decline, especially in elderly patients, and may result in discharge to long-term or rehabilitative care [1]. The levels of mobility (lying, sitting and standing) in hospitalized HF patients are not known. Only one investigator has looked at mobility continuously using accelerometers in the hospitalized patient population [1]. That study was limited to older male patients with a variety of medical diagnoses [1]. Mobility in hospitalized HF patients, who are characterized by a baseline decline in physical function and high rates of hospitalization, has not been described. It is unclear whether accelerometer placement will result in discomfort or skin problems, especially in patients with severe edema. Therefore, the purpose of this pilot study was to describe: 1) the mobility of hospitalized HF patients and the proportion of time they spent standing or walking using accelerometer technology; 2) the physical function in hospitalized HF patients; and 3) the skin condition associated with accelerometer use.

## Methods

### Setting and patients

This was a prospective cohort study that included patients admitted to two telemetry units in a large academic medical center in San Francisco, California. Patients were at least 30 years of age and admitted to either the cardiology or general medical service with a primary or secondary diagnosis of HF between April, 2010 and February, 2011. Because of the need to capture mobility status early during each patients’ hospitalization experience, we included patients with a primary or secondary diagnosis of HF obtained from the electronic medical record. Since not all diagnostic information had been obtained for each patient within the first 24 hours of admission, the diagnosis of HF may not have been known until later during the hospitalization, thus making inclusion of patients with primary or secondary diagnosis of HF necessary. The institutional review board approved this study and all patients provided written informed consent.

Inclusion criteria were: patient-reported ability to ambulate with or without an assistive device during the month prior to hospitalization, an activity order that stated “up ad lib” or allowed the patient to be out of bed, ability to speak English, and no isolation precautions. The latter criterion was based on concerns about the adequacy of sterilization of the accelerometers. Exclusion criteria were: dementia as measured by the MiniCog screening tool [14] or severe dementia noted in the medical record, delirium as measured by the Confusion Assessment Method [15] (CAM), and residence in a skilled nursing facility prior to admission.

### Data collection

Demographic and clinical data were recorded for all subjects using the electronic medical record and demographic data were confirmed by patient interviews. All patients admitted to the study units under either cardiology or medical services within the prior 48 hours were pre-screened daily via chart review. The study procedure consisted of up to 5 hospital visits (Table 1). The study period was initiated by entry into the study within 48 hours of admission and the study end was defined as either day 5 after study entry or the day of hospital discharge, whichever event occurred first. We also recorded the number of medical appliances that were attached to each patient such as oxygen tubing, urinary catheters and intravenous lines. The outcomes of rehospitalization and mortality were followed-up at 90 days after hospital discharge. One graduate nursing student conducted all study procedures and data analysis in collaboration with the principal investigator. The nurse received training regarding equipment and all research instruments.

**Table 1:** Study Procedure

Study Start - Day 1 (Baseline Measures)	Study Days 2 - 4 (Check-in Visits)	Study End - Day 5 or day of discharge (Final Measures)
Demographic: Age, gender, race, marital status; Clinical: Vital Signs, symptoms, Ejection Fraction; Functional: home exercise (self report), NYHA Class, Katz Index of ADLs, KPS, SPPB; Mobility: Accelerometer monitors on ankle and thigh.	Skin: Check skin condition under monitors; Mobility: Move accelerometer monitors to opposite ankle and thigh.	Clinical: Vital Signs, confirm receipt of PT in hospital; Functional: NYHA Class; Skin: Check skin condition under monitors; Mobility: Remove accelerometer monitors.

ADLs: Activities of Daily Living; NYHA: New York Heart Association; KPS: Karnofsky Performance Status Scale; SPPB: Short Physical Performance Battery.

After pre-screening, 103 patients were considered for study approach and 32 were enrolled. Of the 71 patients not enrolled, 46% had a short length of stay (LOS). Short LOS was defined as a discharge planned on the same or next day according to electronic medical record notes, the bedside nurse, or the medical team. Refusals accounted for 27% of those not enrolled. The final 27% were not enrolled for various reasons including having lower extremity skin problems that would interfere with monitor attachment (for example, leg wraps), speaking a language other than English, cognitive problems, transfer off the unit, or not actually being ambulatory.

#### Functional and mobility characteristics

Physical function can be determined by directly measuring performance using previously tested measures such as the Short Physical Performance Battery (SPPB) [16]. Function can also be determined using indirect measures by questionnaire or simply by asking the patient. We assessed physical function directly with the SPPB at the beginning of the study period. Our indirect measures of function included asking the patient about exercise at home (self-report of home exercise) and by questionnaires. Questionnaires included: the New York Heart Association (NYHA) Classification administered at the beginning and end of the study period, the Katz Index of Independence in Activities of Daily Living (ADLs), and the Karnofsky Performance Status scale (KPS).

Function can be measured directly using the SPPB, in which a set of 3 tests (balance time, gait speed, and number of chair stands) are used to objectively measure lower extremity strength. The SPPB was first tested in community-dwelling adults who were 72 years and older as part of the Established Populations for Epidemiological Studies of the Elderly [16]. In the hospital setting, investigators found that the SPPB was safe and feasible, and scores were significantly associated with demographic and clinical variables including increasing age and length of stay [17, 18]. Validity and reliability of the SPPB have been established in the older adult [16].

The NYHA Classification system was first developed in 1928 [19] and describes functional status indirectly using symptoms that range from no symptoms with exertion (NYHA class 1) to symptoms at rest (NYHA class 4) [19]. Although the NYHA class is a subjective measure made by the examiner, studies show that it is valid and reliable [20, 21]. The Katz Index is used to rate six basic activities (dressing, bathing, etc.) [22]. Using a structured questionnaire, we asked participants whether they were independent, required some assistance or total assistance in relation to particular activities. Scoring ranges from zero, denoting complete dependence, to six, indicating that a person is independent when performing ADLs. Validity and reliability of the Katz Index have been established in older adults and in patients after stroke, among others [22, 23]. The KPS is a rating scale based on values between 0 - 100 that assesses the impact of a health condition on the ability to work and care for oneself [24]. Validity and reliability has been established in relation to QOL measures and ADL function in cancer patients, although to our knowledge, our study is the first to use this instrument in hospitalized HF patients [25].

Mobility using accelerometers was measured continuously during the study period via wireless Micro Care Timeliness Monitors (AugmenTech, Inc., Pittsburgh, PA). These are miniature (1 inch square by 0.25 inches depth) devices that record body position using a 3-axis system. These monitors have been previously validated to measure mobility in older hospitalized patients [10]. Two monitors were programmed and then attached to the ipsilateral thigh and ankle of the patient. Each day the skin condition was checked, monitors removed and a new set of programmed monitors was attached to the contralateral ankle and thigh. This occurred daily for up to five days, or until hospital discharge. Positional data relating to three levels of patient mobility (lying, sitting, and standing or walking) was recorded and stored on the monitors. The monitors measure horizontal and vertical position in relation to gravity each second, although the recording interval is programmable. Monitors were set to record data every 4 seconds for this study. The HyperTerminal PE program (Hilgraeve, Inc., Monroe, MI) was used to program monitors and download data. Tegaderm (3M, St. Paul, MN) was used to secure the monitors and gauze pads provided a cushion against the skin.

### Statistical analysis

Data were analyzed using SPSS for Windows software version 19.0 (IBM Corp., Armonk, NY). Descriptive statistics were used to present demographic and clinical characteristics of the patients, including function and mobility. A Student’s t-test was used to compare the time spent while lying, sitting or stand/walking to demographic characteristics. Accelerometer monitor data was processed using Excel (Microsoft, Redmond, WA) and SPSS. Mobility, defined as the mean proportion of time spent in each position (lying, sitting, and standing or walking), was calculated during the study period for each patient using descriptive statistics. Because the accelerometer measures body position in time, the time spent walking versus standing is not discernable. Therefore, time spent walking and standing is grouped together. The date and time of hospital admission was defined as hospital day 1. The start of the study period was determined using the median time from hospital admission. The end of the study period was defined after hospital day 5 or discharge. Daily mobility was determined by taking the mathematical mode of the fifteen 4-second interval positions that comprised each minute, adding up the total minutes spent each position, averaging this over the time on-study for each patient, and then calculating the mean and standard deviation across all study participants. Longitudinal data was calculated based on the total minutes spent in each position on each hospital day (midnight-midnight). Since patients were enrolled and discharged (or finished the study) during the day, 2 study days of mobility data were required to ensure complete data for one full hospital day. Significance was defined as P < 0.05 and data are presented as means ± standard deviations where appropriate.

## Results

### Socio-demographic characteristics

Demographic and clinical characteristics of patients are listed in Table 2. Patients’ mean age was 58 ± 13 with an age range of 30 - 92 years. The majority were male gender (78%, n = 25) and had a history of hypertension (71.9%, n = 23). Most patients smoked in the past (75%, n = 24) and had a mean pack year smoking history of 21. The mean length of stay was 9.5 days, but there was a large standard deviation (± 9.9) indicating a wide range (1 - 41 days) in length of stay. The median length of stay was 6.5 days. Most patients included in the study had HF with reduced ejection fraction or systolic HF (71.9%, n = 23). One-third (34.1%) required physical therapy in the hospital and nearly half (40.6%) were discharged to home with rehabilitation or home health care services. The number of medical appliances that were attached to each patient such as oxygen tubing, urinary catheters and intravenous lines was examined and at least 50% of all patients had at least one attachment. We had a follow-up period of 90 days after hospital discharge and found that 34% of patients had a cardiac readmission and 9% died.

**Table 2:** Sociodemographic and Clinical Characteristics (n = 32)

Characteristic	Value
Age, mean years ± SD	58 ± 13
Male Gender, % (n)	78.1 (25)
Race/Ethnicity % (n)	
Caucasian/White	59.4 (19)
African-American/Black	31.3 (10)
Asian/Pacific Islander	9.4 (3)
History of smoking, % (n)	75.0 (24)
History of hypertension, % (n)	71.9 (23)
Creatinine on admission (g/dL), mean ± SD	1.9 ± 1.7
Etiology of Heart Failure, % (n)	
Ischemic	28.1 (9)
Idiopathic	65.6 (21)
Unknown/other	6.3 (2)
Ejection Fraction < 40%, % (n)	71.9 (23)
ACEi/ARB use - study end, % (n)	62.5 (20)
Beta blocker use - study end, % (n)	78.1 (25)
High-Risk Diagnoses for the Elderly Scale (study end), mean ± SD	
Low (0) = 9.5% chance dying in 1 year	3.3 ± 1.7
Intermediate (1 - 2) = 31% chance	
High risk (3 - 5) = 46% chance	
Very High Risk (> 6) = 74% chance	
Length of hospital stay, mean in days ± SD	9.5 ± 9.9
Physical Therapy - in hospital, % (n)	34.4 (11)
LVAD received during study admission, % (n)	6.3 (2)
Discharged with Physical Therapy, Occupational Therapy, or Home Health, % (n)	40.6 (13)
Cardiac Events - 90 days post-discharge, % (n)	
Cardiac readmission	34.4 (11)
Heart transplant	3.1 (1)
Mortality	9.4 (3)

ACEi: angiotensin converting enzyme inhibitor; ARB: angiotensin receptor blocker; LVAD: left ventricular assistive device.

### Function and mobility characteristics

New York Heart Association (NYHA) class upon enrollment and at the end of the study period improved from class III to class II (mean 2.9 ± 0.8 and 2.2 ± 0.8 respectively) (Table 3). At the start of the study, defined as the allowed enrollment range of 0 - 2 days per inclusion criteria, 18.8% of patients reported symptoms at rest. By the end of the study, a median of 1.0 days prior to discharge, only one patient (3.1%) reported symptoms at rest.

**Table 3:** Functional Status

Characteristic	Value	
Short Physical Performance Battery (mean ± SD) (n = 31)	6.4 ± 3.1	
Accelerometer Mobility (mean minutes and hours ± SD, median minutes and hours)	Mean	Median
Standing or Walking	59 ± 43 minutes	45.8 minutes
Sitting	5.5 ± 3.0 hours	5.8 hours
Lying	16.8 ± 3.2 hours	16.6 hours
Home exercise % (n)	62.5 (20)	
Katz Index of Independence in ADLs (mean ± SD)	5.6 ± 1.1	
NYHA Classification % (n)	Study Start	Study End
Class I	6.3 (2)	15.6 (5)
Class II	18.8 (6)	50.0 (16)
Class III	56.3 (18)	31.3 (10)
Class IV	18.8 (6)	3.1 (1)
Karnofsky Performance Status Scale		
Mean ± SD (range)	71.1 ± 9.0 (50 - 90)	

ADLs: activities of daily living; NYHA: New York Heart Association Class; SD: standard deviation.

The measures of function were varied (Table 3). Most patients reported that they did some form of exercise at home (62.5%) before admission. All patients were ambulatory before admission (per inclusion criteria) and had activity orders that permitted the patient to be out of bed. The mean Katz Index for completion of ADLs upon enrollment was 5.6 ± 1.1 (scores 0 - 6 with 6 best). The mean SPPB scores were 6.4 ± 3.1 (possible scores 0 - 12 with 12 best) with a mean KPS score of 71 ± 9.0 (0 - 100 with 100 independent). Student’s t-tests found that report of exercise at home was significantly related to the average number of hours of lying in bed (P < 0.027).

**Figure 1:**
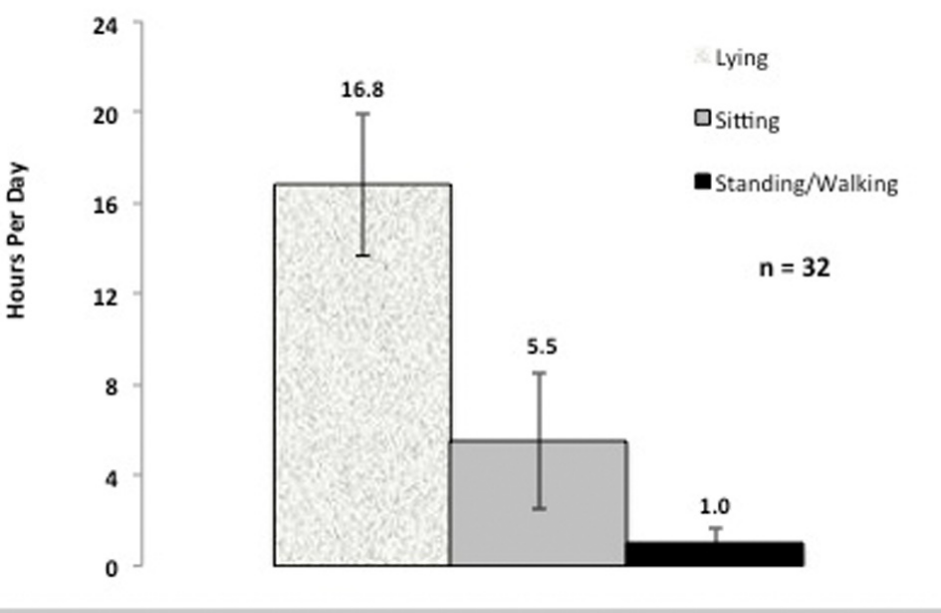
Average daily hours spent lying, sitting, and standing or walking.

**Figure 2:**
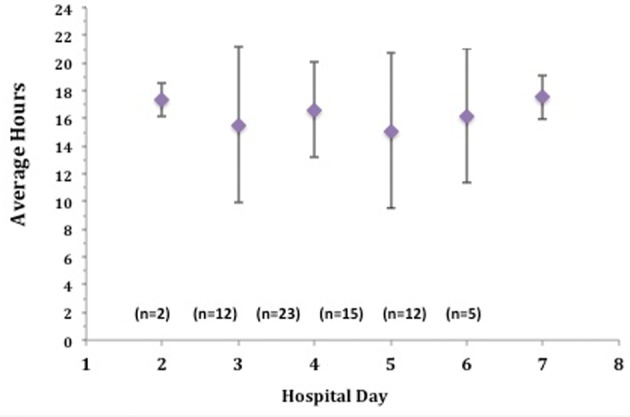
Average daily time spent lying over hospital course.

**Figure 3:**
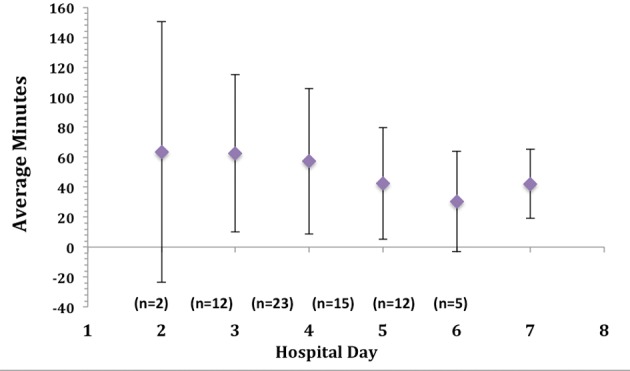
Average daily time spent standing or walking over hospital course.

Accelerometer monitor data during hospitalization revealed that the average time spent standing or walking during a 24 hour time period was 4.1%, or 59 minutes per day (Table 3) (Fig. 1). In order to account for outliers, we also determined the median time for body position. The median time spent standing or walking during a 24 hour time period was 3.2%, or 45.8 minutes per day (Table 3). When looking across subjects, the range of time spent standing or walking during each hospital day was 0-10% (0 - 150 minutes) revealing a limited range of time spent standing or walking. The average time spent lying in bed was 16.8 hours per 24-hour day, or 70% of the measured hospital stay (Table 3) (Fig. 1), while the median time was 16.6 hours or 69% of the measured hospital stay. The average time spent in the study per patient was 3.0 days with a standard deviation of 1.7 days; the median time in the study was 2.7 days. The average daily time spent standing/walking or lying varied little over the patients’ hospital stay (Fig. 2, 3).

Patients were compared by gender in relation to mobility. The mean number of hours females spent lying in bed was 18.4 hours while males spent an average of 16.4 hours lying. The mean number of minutes females spent standing or walking was 41.3 minutes, while males spent an average of 64.3 minutes standing or walking. The mean age of females was 54.9 ± 11.1 years and males were 59.1 ± 14.2 years. Although females were on average younger, they spent more time lying in bed and less time standing or walking than males.

### Skin characteristics

No patient experienced skin irritation that was associated with accelerometer use. All patients reported that the accelerometer was comfortable and did not negatively influence activity or sleep.

## Discussion

This is the first study to describe the amount of time hospitalized HF patients spent lying down, sitting, standing or walking (patient position) using accelerometer technology. The average daily time spent standing or walking was less than one hour (59 minutes) and when median times were examined, even less time was spent standing or walking (45 minutes). Our findings demonstrate that HF patients spent at least 23 hours per day either lying down or sitting, despite a patient-reported ability to ambulate prior to hospitalization, (63%) reporting that they engage in exercise at home, and an “up ad lib” activity order. In fact, one patient had data that suggested that he/she never got out of bed during his/her entire hospital stay. Moreover, the maximum amount of time spent standing or walking each day was 150 minutes or 2.5 hours, demonstrating that the range of physical activity between patients was only 0 - 2.5 hours out of bed per day.

In the only report to describe mobility levels in hospitalized patients using accelerometers, Brown et al. examined the proportion of time spent lying, sitting, standing or walking in hospitalized medical patients [1]. Patients were aged 65 years and older, not demented or delirious, and able to walk in the 2 weeks prior to hospital admission. No patient remained in bed during the entire measured hospital stay, and the median amount of time spent standing or walking was 3% or 43 minutes per day, differing only by 2 minutes from our study. Results from the Brown et al study are strikingly similar to our data despite the difference in age, gender and reason for hospitalization.

In a decade-old study of adults aged 55 and older who were hospitalized for a medical illness, ambulation occurred infrequently with only 27% of patients walking in the hallways during hospitalization [25]. These adults were independent in walking before hospitalization and when ambulating, walked only a median of 5.5 minutes. In a study completed nearly 30 years ago, patients were examined from their preadmission baseline to the second hospital day and 65% of patients experienced a decline in mobility along with significant deterioration in functional score [11]. Although both studies did not use continuous mobility monitoring, the negative outcomes related to continuous immobility among hospitalized patients was elucidated.

The use of accelerometers to provide an accurate measurement of daily activity in HF patients is not reported in the literature at this time. Studies in community dwelling HF patients suggest that activity levels are low, although the measurement methods are not comparable across studies [26-28]. When older adults in the community were examined, results suggest that they are often sedentary [29]. In one study, investigators used accelerometers to measure physical activity levels in adults who were age 70 and older. The average time spent in moderate or vigorous physical activity was just 8.7 minutes for men and 5.4 minutes for women [29]. Despite being sedentary, however, these older adults did not spend the vast majority of their day lying down in bed, as found in our study of hospitalized adults. Further, the mean age of our sample was much younger at 58 years. Although the American Heart Association guidelines for HF management recommend that exercise training should be considered for all stable HF patients, optimal exercise protocols for HF patients do not exist [30]. Results from the HF-Action study, a large (n = 2,331) randomized controlled trial that included stable outpatients with HF, do provide evidence that exercise most days of the week does decrease rates of all cause hospitalizations or death [31]. All patients in this study had low ejection fraction HF.

Results from our study also demonstrated that men spent more time walking or standing and less time lying down than women despite their older mean age. Fisher and colleagues used a pedometer to measure steps in older hospitalized patients in two studies [32, 33]. In one study gender was not associated with activity during hospitalization in older adults [33], while in another gender was associated with a change in step score; for example, men increased the number of steps walked during their hospitalization more than women [32]. As noted above, in a study of older community dwelling adults, men spent more time in moderate to vigorous activity than women [29]. However, our sample size of females is small and needs to be interpreted with caution.

The mean SPPB scores of 6.4 ± 3.1 (possible scores 0 - 12) that were measured upon enrollment into the study revealed low physical function levels. However, the mean Katz Index, a measure that uses self-report, of 5.6 ± 1.1 (upon enrollment) demonstrated minimal dependence on assistance for completion of ADLs (possible scores 0 - 6). The discrepancy between the SBBP and Katz could be explained by the fact that HF patients may have overestimated their level of function through self-report. Or, they may be able to perform their activities of daily living despite the poor lower extremity strength that was found by directly measuring their function (SPPB). The mean Karnofsky Performance Status score was 71 ± 9.0, indicating that the average patient was unable to work but could live at home and take care of most of their daily needs.

A growing appreciation for the benefits of early mobility can be seen in studies involving intensive care unit (ICU) patients. Investigators have reported data on the benefits of early ambulation in the chronically critically ill adult [34]. Twice daily, rehabilitation therapy in 103 mechanically ventilated patients demonstrated that 69% of patients were ambulating more than 100 feet by ICU discharge. A larger ICU study compared an early mobility intervention with a usual care group and found that patients who were mobilized during their ICU stay had a shorter length of stay in the ICU and the hospital [35].

Low mobility can have multiple direct and indirect effects on musculature in patients with HF. In addition to underlying skeletal muscle atrophy coupled with worsening cardiac function, low mobility may be associated with poor outcomes such as hospital readmission in relation to cardiac function, but may also be related to non-cardiac admissions such as falls. However, low mobility and related outcomes has yet to be directly studied in patients with HF. In older hospitalized medical patients, hospital stays have been associated with loss of independence and physical decline, and may result in discharge to long-term or rehabilitative care rather than home [1, 17, 36].

The hospital environment is often not conducive to ambulation. We found that more than 56% of the patients had at least 1 medical appliance attached (range 0 - 2). Items such as oxygen tubing, urinary catheters and intravenous lines “tether” the patient and provide opportunities for falls or injuries and the need for assistance during ambulation. Just one appliance usually requires some staff support for getting out of bed, thereby setting up a barrier for increasing mobility. In many hospitals, the patients’ small television is mounted on a swinging arm, allowing the patient to simply raise their arm to bring the television into view. Without a destination of interest such as a patio, garden, or entertainment center, the incentive to ambulate may not be present. The old medical order “up ad lib,” meaning “getting up without restraint or imposed limit,” also provides a subtle message of acceptable confinement: it permits rather than actively encourages patients to get out of bed, and does not provide any guidance regarding the recommended frequency, duration, or distance of ambulation [37]. Surgical patient may populations have different activity orders to specify when and how often they should get out of bed after their procedure, with a strong focus on avoiding the complications that occur with prolonged bedrest following surgery. Patient symptoms may also play a role in mobility since activity-limiting levels of dyspnea and fatigue are often present in HF patients. However, NYHA class was not associated with length of time lying in bed or with time standing or walking in our study. In fact, mean NYHA class did improve from our study start to study end (2.9 study start, 2.2 study end), but time spent standing or walking did not increase over the hospital stay (Fig. 3). This finding is not consistent with findings in a study of older hospitalized patients. Fisher and colleagues examined ambulation in older adults using pedometers [33]. The study took place on an Acute Care for Elderly unit which emphasizes ambulation. Patients with shorter lengths of stay tended to increase ambulation on day 2 of hospitalization. In our study, we had no appreciable increase in ambulation over the hospital stay as patients approached their day of discharge.

### Strengths and limitations

This was a pilot study with a small sample size, thereby limiting generalizability. The sample included more men than women, providing a skewed distribution in gender. The sample did not include patients that were hospitalized for short stays (less than 48 hours) or for observation so this data cannot be applied to short stay patients. Nonetheless, this study is the first to monitor levels of mobility in hospitalized HF patients continuously using an objective measurement with accelerometers and provides a beginning understanding of mobility patterns in this population. Finally, the accelerometers used were previously validated and proved to be an unobtrusive means of gathering objective data on mobility.

### Conclusion

Immobility has numerous adverse effects such as muscle atrophy, deconditioning, and functional decline. Heart failure patients in particular have both cardiac muscle and skeletal muscle dysfunction, and when hospitalizations occur, outcomes such as functional decline and mortality are not known in relation to mobility. Using new technology, we found that hospitalized HF patients spent a mean of 59 and a median of 45 minutes standing or walking daily, with almost their entire hospital stay spent sitting or lying in bed. This sedentary pattern occurred despite patients being ambulatory prior to their hospitalization. Future studies to examine outcomes such as hospital readmission in relation to mobility status are needed.
